# Lower Limb Serratia marcescens Necrotizing Fasciitis Complicated by Nosocomial COVID-19

**DOI:** 10.7759/cureus.33453

**Published:** 2023-01-06

**Authors:** Raiyyan Aftab, Jing Qin Tay, Philip Sauvé, Ankur Pandya, Zhi Yang Ng

**Affiliations:** 1 Plastic Surgery, Queen Alexandra Hospital, Portsmouth, GBR; 2 Plastic Surgery, Salisbury District Hospital, Salisbury, GBR; 3 Orthopedics, Queen Alexandra Hospital, Portsmouth, GBR

**Keywords:** nosocomial infections, serratia, lower limb, covid-19, necrotizing fasciitis (nf)

## Abstract

*Serratia marcescens* represents an unusual yet potentially deadly cause of lower limb necrotizing fasciitis (NF). Compounding the already high mortality of NF, *S. marcescens* infections are usually associated with worse outcomes (i.e., amputation). Here we present the case of a 56-year-old immunocompromised man due to lupus nephritis who developed lower limb NF secondary to *S. marcescens *followed by nosocomial coronavirus disease 2019 (COVID-19) pneumonitis. Successful limb salvage was achieved through a multidisciplinary team approach from various specialties including plastic surgery, orthopedic surgery, anesthesiology, intensive care, respiratory medicine, and nephrology. At 11 months' follow-up, the patient was largely independent with activities of daily living and was able to ambulate. Unfortunately, he suffered a myocardial infarction at 19 months post-operatively and passed away. A review of the literature revealed only a handful of cases of lower limb NF due to *S. marcescens *and none with subsequent COVID-19. Therefore, this is the first report of such a case which should help with the clinical management of such cases going forward, especially with COVID-19 now becoming endemic in our communities and contributing to delayed presentations and increased mortality in NF.

## Introduction

*Serratia marcescens* is a Gram-negative, opportunistic nosocomial pathogen that is a part of the Enterobacteriaceae family. Reservoirs of the pathogen can be found in the aerodigestive, respiratory, and urinary tracts [1]. It is associated with infections within the aforementioned systems and can present as a rare and deadly cause of necrotizing fasciitis (NF). Globally, there have only been about 15 reported cases of lower limb NF due to *S. marcescens* with reportedly worst outcomes - 52.1% mortality, compared with 20.6% for NF overall [2,3]. Risk factors identified for NF secondary to *S. marcescens* include immunosuppression, renal failure with dialysis dependence, and diabetes [4], which is not dissimilar to NF caused by other organisms [5]. Since the first case report describing *S. marcescens *related lower limb NF in 1987, its reported incidence has been slowly increasing [6]. Furthermore, the literature represents a bleak view of these cases of lower limb NF caused by *S. marcescens* - up until 2016, only two cases out of nine have managed to survive without amputation of the affected lower limb with the remaining patients succumbing to the disease [7]. This is likely secondary to the fulminant course of NF caused by *S. marcescens*. Here we present the first reported case of lower limb NF secondary to *S. marcescens* complicated by the development of nosocomial coronavirus disease 2019 (COVID-19) pneumonitis and review the clinical management during this most challenging of times.

## Case presentation

A 56-year-old male with end-stage renal disease (ESRD) secondary to lupus nephritis and renal cell carcinoma presented with a 24-hour history of left medial knee pain and bruising without any history of trauma. On examination, he was septic with vitals of 38.3°C, blood pressure (BP) of 89/57 mmHg, and heart rate (HR) of 110 beats per minute. There was a patch of purplish discoloration extending from the left mid-tibial region to the left medial knee, but without any overlying blisters. This was tender on palpation but there were no clinical signs of joint effusion or subcutaneous crepitus. The neurovasculature of the affected left lower limb was intact distally. Review of systems was otherwise unremarkable. His current medications include prednisolone, famotidine, erythropoietin, ramipril, alfacalcidol, levetiracetam, amlodipine, bisoprolol, ticagrelor, folic acid, aspirin, renal multivitamins, and tadalafil. Initial workup showed hemoglobin (Hb) 10.8 g/dL, white blood cell count (WCC) 4.4x10^9^/L, platelet 39x10^9^/L, c-reactive protein (CRP) 99 mg/L, and lactate 9 mmol/L; the laboratory risk indicator for necrotizing fasciitis (LRINEC) score was 4 (Table 1).

**Table 1:** Results from initial laboratory work-up. LRINEC: laboratory risk indicator for necrotizing fasciitis; eGFR: estimated glomerular filtration rate

Test	Result
Hemoglobin	108 g/L
Hematocrit	0.33 L/L
WBC	7.9x10^9^/L
Neutrophils	7.8x10^9^/L
Lymphocytes	0.2x10^9^/L
Monocytes	0.6x10^9^/L
Eosinophils	0.0x10^9^/L
Basophils	0.0x10^9^/L
Platelets	39x10^9^/L
Urea	20.4 mmol/L
Sodium	138 mmol/L
Creatinine	943 µmol/L
Glucose	4.7 mmol/L
eGFR	0 mL/min/1.73 m²
C-reactive protein	99 mg/L
Lactate	9 mmol/L
LRINEC score	4 (hemoglobin <110 g/dL, creatinine >141 µmol/L; +2 each)

The working diagnosis, however, was that of NF of the left lower limb. Hence, the patient was commenced on IV piperacillin-tazobactam, clindamycin, and vancomycin as per our institution’s guidelines. A platelet transfusion was also given in preparation for emergent surgical debridement, which was performed within about 4 hours of hospital arrival under general anesthesia despite the patient's known history of malignant hyperthermia. Intra-operatively, there was significant tissue necrosis and edema in the fascial planes. Two further units of packed red cells and platelets and additional vasopressor support were required. The final post-debridement defect had near circumferential involvement from the knee down to just above the ankle joint. This was dressed temporarily with a negative pressure vacuum-assisted closure (VAC) device. A relook debridement the next day required only minimal further excision. Post-operatively, the patient was managed in the ICU for 12 days during which he received three days of IV metronidazole and 10 days of IV meropenem as per microbiological sensitivities (Table 2).

**Table 2:** Serratia marcescens antibiotic sensitivity profile.

*Serratia marcescens *microbiology samples and results	
Sites isolated	Wound swabs, knee aspirate, tissue samples, and blood cultures
Resistant	Amoxicillin, co-amoxiclav, chloramphenicol, ertapenem, cefoxitin, gentamicin, and piperacillin-tazobactam
Sensitive	Ciprofloxacin, co-trimoxazole, and meropenem

**Figure 1:**
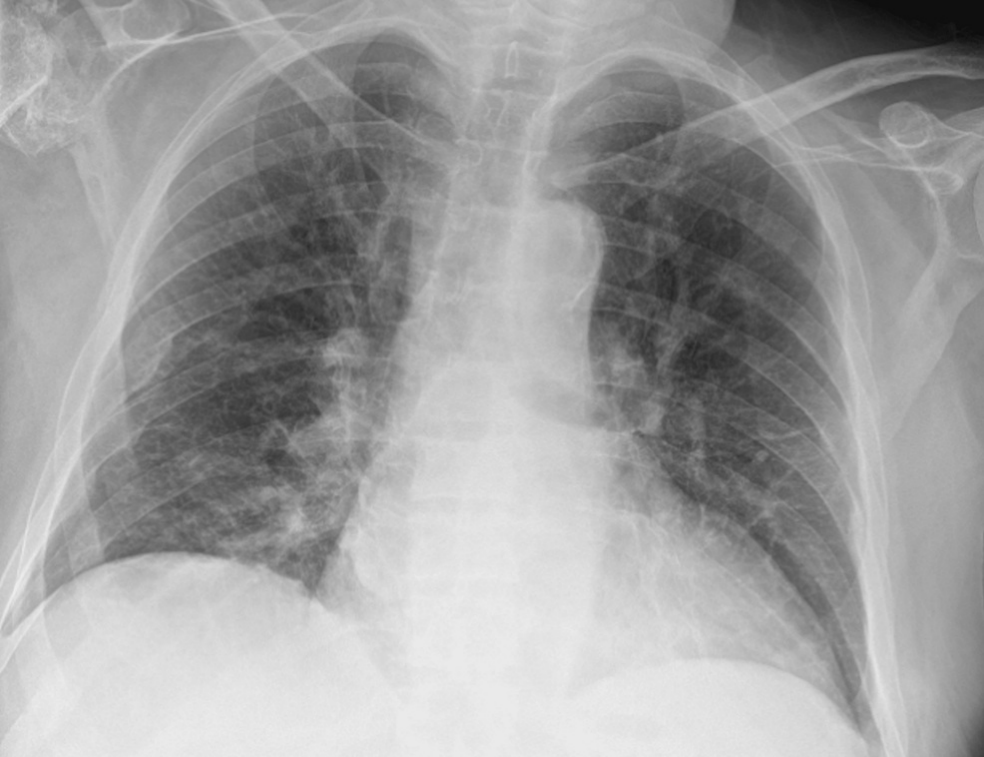
AP chest x-ray showing right middle zone consolidation, in keeping with pneumonia. AP: anteroposterior

He was then stepped down to the general ward with plans for VAC dressing changes in the OR. The aim was to allow further granulation of the post-debridement left lower limb wounds, in view of his extensive co-morbidities that may impair wound healing such as immunosuppression and ESRD, prior to definitive coverage with skin grafting. However, he developed nosocomial COVID-19 pneumonitis with shortness of breath and tachypnea at 32 days following his initial surgery (at 20 days on the ward). This was confirmed with a polymerase chain reaction (PCR) test. He required supplemental oxygen, 10 days of PO dexamethasone as well as IV co-amoxiclav to cover for superimposed bacterial pneumonia (Figure 1).

**Figure 2:**
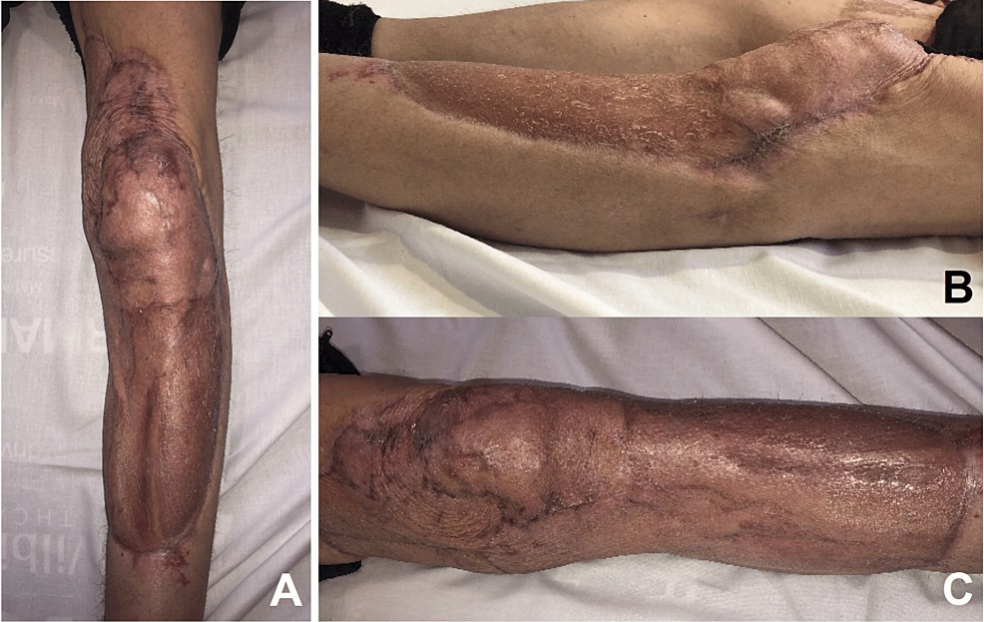
Frontal (A), lateral (B), and medial (C) views of the left lower limb following reconstruction with split-thickness skin graft at 11 months post-operative, with good contour, reasonable cosmesis, and absence of fixed flexion deformities and contractures at the knee joint.

All this occurred during the peak of the COVID pandemic in the United Kingdom (October to December 2020). This, in addition to the patient’s history of malignant hyperthermia, led to a clinical decision to further delay wound closure by continuing on twice-weekly VAC dressing changes until his PCR test was negative for COVID-19. Finally, on day 63, the patient underwent skin grafting with additional back slab and VAC dressings under spinal anesthesia. Wound check a week later revealed 100% graft take and he was discharged a few days later. The patient was last seen at 11 months out with no contractures or wound breakdown (Figures 2A-2C).

He was otherwise ambulant and largely independent with activities of daily living. Unfortunately, he passed away 19 months after his initial surgery due to a myocardial infarction.

## Discussion

Globally, NF is associated with significant mortality and this is further increased when caused by atypical organisms such as *S. marcescens* [2]. A recent study by Nguyen et al. showed that in Australia, patients with NF during the COVID-19 lockdown tended to present later and more critically ill, leading to a 50% mortality rate compared to 0% in their pre-COVID-19 cohort [8]. Of note, none of the patients in the study actually tested positive for COVID-19 (they were distinguished by study period only, i.e., pre- and during COVID-19). Therefore, this further reiterates the challenges that we were faced with in the current case - an immunosuppressed patient presenting with a rare form of lower limb NF caused by *S. marcescens*, which was then complicated further by the development of nosocomial COVID-19 pneumonitis post-operatively.

While studies such as that by Nguyen et al. suggest that the mortality rate of NF during COVID-19 was higher, this is likely due to further progression of pathology due to delayed presentation (mean of 4.1 days) as demonstrated by more highly deranged biochemical results (i.e., doubling of creatinine and lactate, and tripling of urea) [8]. In turn, the LRINEC score in these patients was an average of 5.9, which equates to an intermediate (50-75%) risk of developing NF. By comparison, our patient presented within 24 hours with an LRINEC score of 4, which suggested a low risk for developing NF. Ultimately, this was not the case and highlights the importance of having a low index of clinical suspicion for NF, particularly when there are significant risk factors such as long-term immunosuppression. Of note, lower limb infections with *S. marcescens* have been reported to present variably with cellulitis, eschar formation, and bullae formation. In all likelihood, the true incidence of lower limb *S. marcescens* is most certainly underestimated and the severity of infection presents along a spectrum with NF obviously the most lethal [9].

The development of nosocomial COVID-19 pneumonitis post-operatively, on top of the patient’s history of malignant hyperthermia, led to our decision to delay skin grafting of the wound for almost four weeks with bi-weekly VAC changes on the ward. Fortunately, this patient was vaccinated against COVID-19, so he was able to make an otherwise uneventful recovery after treatment with steroids and antibiotics. Following reconstruction with skin grafts, the patient was able to return to >50% of his baseline function at 11 months out. Unfortunately, the patient passed away at 19 months post-operatively due presumably to an unrelated myocardial infarction although we cannot completely rule out it being late sequelae of COVID-19 and its various cardiac manifestations, such as stress cardiomyopathy.

## Conclusions

Lower limb NF due to *S. marcescens* represents a potentially highly lethal combination. With COVID-19 now considered endemic, it is but a matter of time before more cases of NF due to atypical bacteria with concurrent or subsequent (as with this case) COVID-19 present. In fact, COVID-19 has also been implicated as the cause of NF itself in certain cases. At the same time, for a patient to contract an atypical bacterial infection, it means that he is also likely to be more susceptible to contracting COVID-19. Further study of the potential synergy (or not) between COVID-19 and atypical bacteria in the pathogenesis of NF will therefore be required.
